# A Narrative Review of Scoring Methods in Disseminated Intravascular Coagulation: Evaluating Diagnostic Accuracy and Clinical Utility

**DOI:** 10.7759/cureus.67052

**Published:** 2024-08-17

**Authors:** Pulivarthi Chaithanya, Revat J Meshram, Amar Taksande

**Affiliations:** 1 Pediatrics, Jawaharlal Nehru Medical College, Datta Meghe Institute of Higher Education and Research, Wardha, IND

**Keywords:** jaam dic criteria, isth criteria, clinical utility, diagnostic accuracy, scoring systems, disseminated intravascular coagulation (dic)

## Abstract

Disseminated intravascular coagulation (DIC) is a critical, life-threatening disorder characterized by widespread activation of the coagulation cascade, leading to microthrombi formation, consumption of clotting factors and platelets, and a paradoxically increased risk of bleeding. Accurate and timely diagnosis is crucial for effective management and improved patient outcomes. This narrative review aims to evaluate the diagnostic accuracy and clinical utility of various scoring systems used to assess DIC. We examine prominent systems, including the International Society on Thrombosis and Haemostasis (ISTH) scoring system, the Japanese Association for Acute Medicine (JAAM) DIC criteria, and other regional or institutional criteria such as the Chinese DIC scoring system (CDSS). The review compares these systems based on their criteria, sensitivity, specificity, and accuracy across different patient populations and discusses their strengths and limitations. Additionally, we explore the impact of these scoring systems on patient management and therapeutic decisions, identify challenges and limitations, and highlight emerging trends and future directions in DIC diagnosis. By providing a comprehensive analysis, this review aims to enhance understanding of DIC scoring methods and inform clinical practice to improve patient care.

## Introduction and background

Disseminated intravascular coagulation (DIC) is a severe and complex disorder characterized by the widespread activation of the coagulation cascade. This activation results in the formation of microthrombi throughout the small blood vessels, consuming clotting factors and platelets and creating a paradoxical risk of bleeding [1]. DIC can arise secondary to a variety of underlying conditions, including sepsis, trauma, malignancy, and obstetric complications. Its systemic nature and potential to rapidly progress to life-threatening complications underscore the critical need for accurate diagnosis and timely intervention [2]. The importance of an accurate and timely diagnosis of DIC cannot be overstated. Early recognition allows for prompt treatment of the underlying cause, which is essential for managing the coagulation disorder and improving patient outcomes. Delay in diagnosis or misdiagnosis can lead to severe consequences such as organ dysfunction, increased bleeding risks, and higher mortality rates. Thus, reliable diagnostic tools and scoring systems are indispensable for clinicians to identify and manage DIC effectively [3].

Several scoring systems have been developed to assist in diagnosing and assessing DIC. These systems utilize a combination of clinical and laboratory parameters to gauge the severity of the disorder and inform treatment decisions [4]. The International Society on Thrombosis and Haemostasis (ISTH) scoring system is one of the most recognized, employing a set of criteria to classify the severity of DIC. Another notable system is the Japanese Association for Acute Medicine (JAAM) DIC criteria, specifically designed for acute medical conditions and widely used in clinical practice in Japan. Additionally, other regional or institutional criteria, such as the Chinese DIC scoring system (CDSS), offer variations tailored to specific patient populations and settings [5]. This review aims to evaluate and compare the diagnostic accuracy and clinical utility of these various DIC scoring systems. By examining the strengths and limitations of each system, the review aims to provide a comprehensive understanding of their effectiveness in clinical practice. Furthermore, this review will explore emerging trends and future directions in DIC diagnosis to enhance patient care and optimize clinical outcomes.

## Review

Pathophysiology of DIC

DIC is a complex syndrome marked by systemic activation of the coagulation cascade, leading to the formation of microvascular thrombi and potential bleeding complications. The pathophysiology of DIC involves several interconnected mechanisms, primarily driven by the dysregulation of coagulation factors, fibrinolysis, and endothelial dysfunction [6]. DIC often begins with the exposure of tissue factor (TF) to circulating blood, which can occur due to various pathological conditions such as sepsis, trauma, or malignancy. TF, a procoagulant glycoprotein, binds to activated factor VII, forming a complex that activates factors IX and X, ultimately leading to increased thrombin generation [3]. This excessive thrombin promotes the conversion of fibrinogen to fibrin, resulting in widespread clot formation within the microvasculature. As DIC advances, clotting factors and platelets are consumed, which can cause a paradoxical bleeding tendency despite the presence of thrombi. This consumption is further exacerbated by the ongoing activation of the coagulation cascade, leading to a clinical presentation characterized by both thrombosis and hemorrhage [6].

Fibrinolysis, the process of breaking down fibrin clots, is also significantly altered in DIC. The balance between coagulation and fibrinolysis becomes disrupted, leading to insufficient fibrin degradation or accelerated fibrinolysis. In some cases, the release of fibrin degradation products, such as D-dimer, can be markedly elevated, indicating intense fibrinolytic activity. However, large amounts of fibrin can overwhelm the fibrinolytic system, contributing to the formation of microthrombi and subsequent organ dysfunction. This duality, where both clot formation and breakdown are dysfunctional, complicates the clinical management of DIC [7]. Endothelial cells play a crucial role in maintaining hemostatic balance, and their dysfunction is a significant factor in the pathophysiology of DIC. In this condition, endothelial dysfunction arises from the inflammatory response associated with the underlying disease process. This dysfunction can lead to increased procoagulant factors and decreased expression of anticoagulant factors [8]. The loss of endothelial integrity facilitates the exposure of tissue factors to the bloodstream and promotes thrombosis. Additionally, inflammatory mediators released during this process can further exacerbate endothelial injury, creating a vicious cycle of coagulation and inflammation [9].

Clinical presentation and diagnostic challenges

DIC presents a complex array of clinical manifestations and diagnostic challenges due to its multifaceted nature and the variability of symptoms depending on underlying conditions. The clinical presentation of DIC can be highly variable, but common manifestations include bleeding, thrombosis, and organ dysfunction [8]. Patients with DIC often experience bleeding from multiple sites, including the gums, gastrointestinal tract, and intravenous lines. Petechiae and ecchymosis are frequently observed, especially in acute cases. Although bleeding is a prominent feature, thrombosis can also occur, leading to complications such as organ dysfunction. This is due to the simultaneous activation of coagulation pathways, which can result in microvascular thrombi and ischemia in various organs. Symptoms of organ failure may emerge, including renal dysfunction (with up to 25% of patients presenting with renal failure), respiratory issues (such as dyspnea and hemoptysis), and neurological symptoms (such as altered mental status or focal neurological deficits due to cerebral ischemia) [10]. The symptoms of DIC often overlap with those of the underlying disorder, whether it be sepsis, trauma, malignancies, or obstetric emergencies. For instance, in sepsis, systemic inflammatory responses may worsen clotting abnormalities.

Diagnosing DIC is challenging due to the absence of a definitive gold standard test, the subtlety of clinical signs in chronic or smoldering cases, and the potential overlap with other conditions. Interpretation of scoring systems also adds to the complexity [1]. No single laboratory test can definitively diagnose DIC. Instead, diagnosis relies on clinical evaluation and laboratory findings, such as prolonged clotting times, thrombocytopenia, and elevated fibrin degradation products. Clinical and laboratory abnormalities may be subtle in chronic or smoldering cases, complicating the diagnosis. Symptoms may not indicate DIC, leading to potential misdiagnosis or delayed treatment. Additionally, DIC symptoms can overlap with other coagulopathies and conditions that cause bleeding or thrombosis, such as liver disease or other hematological disorders, necessitating a comprehensive evaluation of the patient's clinical picture and history [11].

**Figure 1:**
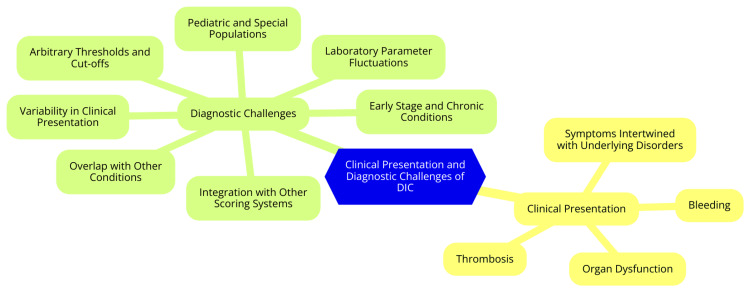
The clinical presentation and diagnostic challenges of disseminated intravascular coagulation (DIC) Image credit: Dr. Pulivarthi Chaithanya

Various scoring systems have been proposed to aid diagnosis, including those developed by the ISTH and the Japanese Society on Thrombosis and Hemostasis (JSTH). However, these systems require careful interpretation and depend on the availability of specific laboratory tests. In summary, the clinical presentation of DIC is complex, involving both bleeding and thrombotic events, which complicates its diagnosis. Clinicians must remain vigilant in recognizing the signs of DIC and use a combination of clinical judgment and laboratory assessments to achieve an accurate diagnosis [12]. The clinical presentation and diagnostic challenges of DIC are shown in Figure 1.

Current scoring systems for DIC

DIC is a complex and critical condition requiring precise diagnosis for effective management. Several scoring systems have been developed to assist in identifying DIC, with the most prominent being the ISTH scoring system, the JAAM DIC criteria, and various regional or institutional criteria, such as the CDSS [1]. The ISTH scoring system is widely recognized for diagnosing overt DIC. It is based on four key laboratory parameters: platelet count, prothrombin time (PT), fibrinogen level, and fibrin degradation products (such as D-dimer). Each parameter is assigned a specific point value, and a total score of 5 or more indicates the presence of overt DIC. This system is precious as it integrates clinical and laboratory findings, enabling a comprehensive assessment of the patient's coagulation status. The ISTH criteria are known for their high sensitivity and specificity, making them a reliable tool in clinical practice [2].

In contrast, the JAAM DIC criteria are designed for specific clinical settings, particularly acute medicine. This scoring system categorizes DIC into three types based on the underlying pathology: basic, hematopoietic, and infectious. Each category has distinct scoring thresholds, with a total score of 6 or more indicating DIC for fundamental causes and four or more for hematopoietic causes. The JAAM criteria are beneficial in emergencies where rapid assessment is crucial [5]. Various regional or institutional criteria have also been developed to address specific clinical needs. For instance, the CDSS has been tailored to reflect China's unique patient populations and healthcare contexts. While the specifics of this scoring system may vary, it generally includes laboratory parameters and clinical findings similar to those of the ISTH and JAAM systems. Modified versions of the ISTH score have also been proposed to suit resource-limited settings, often omitting specific parameters such as fibrinogen levels to simplify the diagnostic process while maintaining accuracy [13]. The current scoring systems used for diagnosing and assessing DIC are shown in Table 1.

**Table 1:** Current scoring systems used for diagnosing and assessing disseminated intravascular coagulation (DIC)

Scoring System	Organization	Criteria	Score Interpretation	Comments
ISTH Scoring System	International Society on Thrombosis and Haemostasis (ISTH)	1. Platelet count <100 x 10^9^/L 2. Elevated fibrin-related markers (D-dimer, FDP) 3. Prolonged PT (prothrombin time) 4. Fibrinogen level <1 g/L	≥ 5: Overt DIC < 5: Non-overt DIC	Widely used; requires laboratory tests; used for both acute and chronic DIC
JAAM Scoring System	Japanese Association for Acute Medicine (JAAM)	1. Platelet count <120 x 10^9^/L 2. Elevated fibrin degradation products (FDP) ≥25 μg/mL 3. Prolonged PT ratio ≥1.2 4. Systemic inflammatory response syndrome (SIRS) present	≥ 4: DIC positive	Focuses on sepsis-related DIC; higher sensitivity for early detection
KSTH Scoring System	Korean Society on Thrombosis and Hemostasis (KSTH)	1. Platelet count <100 x 10^9^/L 2. Elevated D-dimer or FDP 3. Prolonged PT ≥3 seconds 4. Fibrinogen level <1.5 g/L	≥ 4: DIC positive	Similar to ISTH but includes a different threshold for fibrinogen
MHLW Scoring System	Ministry of Health, Labour and Welfare (Japan)	1. Platelet count <80 x 10^9^/L 2. Elevated FDP ≥10 μg/mL 3. Prolonged PT ≥3 seconds 4. Fibrinogen level <1.0 g/L	≥ 3: DIC positive	Primarily used in Japan; focuses on sensitivity for early DIC detection
Chinese DIC Scoring System	Chinese Society of Hematology	1. Platelet count <100 x 10^9^/L 2. Elevated D-dimer or FDP 3. Prolonged PT ≥3 seconds 4. Fibrinogen level <1.0 g/L	≥ 5: DIC positive	Similar to ISTH with modifications to suit the Chinese population
MODS-DIC Score	Multi-Organ Dysfunction Score (MODS)	1. Organ failure (respiratory, renal, hepatic) 2. Hemodynamic instability 3. Thrombocytopenia	No specific threshold; part of a broader assessment of organ dysfunction	Used to assess DIC in the context of multi-organ dysfunction

Comparison of scoring systems

When comparing scoring systems for DIC, it is crucial to assess their criteria, sensitivity, specificity, accuracy, and the advantages and limitations of each system. The most commonly used scoring systems include the ISTH, Japanese Ministry of Health and Welfare (JMHW), JAAM, and CDSS [14]. The ISTH scoring system is one of the most widely recognized and utilized frameworks for diagnosing DIC. It incorporates four key laboratory parameters: platelet count, PT, fibrinogen levels, and fibrin-related markers such as D-dimer. Each parameter is assigned a score, with a total score of 5 or more indicating overt DIC. The ISTH system has demonstrated diagnostic solid performance, with sensitivity reported at 93% and specificity at 98%, making it particularly effective in critically ill patients [15]. In contrast, the JMHW scoring system also emphasizes laboratory findings but places greater importance on clinical features and specific thresholds for test results. Designed primarily for use in Japan, this system accounts for the underlying disease context and shows effectiveness in clinical scenarios, particularly within Japanese populations. However, its sensitivity and specificity can vary, and it is less recognized outside Japan [3].

The JAAM scoring system is tailored for acute clinical settings and integrates clinical assessment and laboratory results. It employs a scoring approach similar to the ISTH but specifically designed for emergencies. The JAAM system has been validated in various studies, showing good sensitivity and specificity, especially in acute conditions. Nevertheless, its applicability may be limited in chronic cases or diverse populations [16]. The CDSS is a newer scoring system developed for the Chinese population, combining clinical manifestations with laboratory results. It focuses on organ dysfunction and D-dimer levels, with scoring thresholds determined based on early patient enrollment. Preliminary validation of the CDSS has shown promising results, with sensitivity and specificity comparable to the ISTH system, particularly in diverse patient populations. However, further validation is needed to confirm its broader applicability beyond China [13].

Each scoring system has its strengths and limitations. The ISTH system is widely accepted and validated, making it a reliable choice, although it may be less applicable in non-critically ill patients. The JMHW system improves diagnostic accuracy by incorporating clinical context but is less known outside Japan. The JAAM system is relevant for emergencies but may lack validation in chronic conditions. Lastly, while the CDSS shows potential in predicting outcomes and integrating local clinical practices, it requires additional validation for broader use. The choice of scoring system should be guided by the specific clinical setting and patient population, with ongoing evaluation needed to enhance diagnostic accuracy and patient outcomes in DIC [5].

Clinical utility and impact on patient management

Scoring systems are essential in diagnosing and managing various clinical conditions, including DIC. They provide a standardized method for assessing disease severity, predicting outcomes, and guiding therapeutic decisions by quantifying clinical and laboratory parameters [4]. For example, the ISTH criteria for DIC use laboratory results such as platelet count, PT, and fibrinogen levels to assign scores, indicating the presence and severity of the condition. This quantification supports early diagnosis and timely intervention, crucial for managing a rapidly evolving disorder such as DIC [17]. Beyond diagnosis, scoring systems enhance prognostic accuracy by correlating scores with patient outcomes [18]. Systems such as the Acute Physiology and Chronic Health Evaluation (APACHE) and the Sequential Organ Failure Assessment (SOFA) are commonly used in intensive care settings to predict mortality and morbidity based on initial assessments of a patient's physiological status. Clinicians can tailor management strategies more effectively by stratifying patients according to risk scores. Higher scores may necessitate more aggressive treatment approaches, while lower scores might suggest a more conservative management strategy. This stratification is particularly valuable in conditions such as DIC, where balancing the risks of bleeding and thrombosis is critical [19].

Scoring systems also impact therapeutic decisions by indicating the need for intensive therapies. For instance, a high DIC score may prompt immediate interventions such as administering anticoagulants or blood products to manage coagulopathy and prevent complications. Additionally, scoring systems assist in resource planning and allocation in critical care settings by providing insights into the expected workload and resource needs based on patient severity scores. This is crucial for effective management in high-demand environments such as intensive care units (ICUs) [20]. Moreover, repeated assessments using scoring systems allow ongoing evaluation of a patient's response to treatment. Changes in scores can signal the need to adjust therapeutic approaches, ensuring that management remains aligned with the patient's evolving clinical status. In summary, scoring systems are integral to clinical practice, enhancing diagnostic accuracy, guiding therapeutic decisions, and improving patient management outcomes. Their application not only aids in individual patient care but also supports broader resource management in healthcare settings, ultimately contributing to better patient outcomes [21].

Limitations and challenges

The limitations and challenges of scoring systems for DIC are diverse and can impact their clinical utility and performance. A primary challenge is the variability in clinical presentation. DIC can manifest differently depending on the underlying condition, such as sepsis, trauma, or obstetric complications, leading to inconsistent scoring outcomes. Many scoring systems may not fully account for these diverse clinical scenarios, affecting their reliability and accuracy [20]. Another challenge is the dependence on laboratory parameters such as platelet counts, PT, and fibrinogen levels. Variability in these parameters, influenced by factors such as sample handling, test timing, and the patient's status, can impact the accuracy of the scores. For instance, fluctuations in laboratory values can lead to discrepancies in the interpretation of the severity of DIC [22]. The thresholds and cutoffs used in scoring systems can also be arbitrary and not universally applicable. For example, a platelet count of less than 50 x 10^9^/L might indicate severe DIC in one population but not in another, leading to potential misclassification of disease severity. Moreover, integrating DIC scores with other scoring systems, such as the SOFA, can enhance prognostic accuracy and complicate interpretation and clinical decision-making, particularly in acute settings [23].

In the early stages of DIC, laboratory markers may not yet show significant changes, potentially leading to underdiagnosis. This delay can result in scoring systems failing to capture early signs of DIC, thereby delaying necessary treatment. Similarly, standard laboratory values may be altered in patients with chronic conditions or those undergoing anticoagulation therapy, complicating the application of conventional scoring systems and potentially leading to misinterpretation of disease severity [3]. Special populations, such as pediatric patients or those with unique clinical scenarios, also present challenges. Scoring systems primarily validated for adults may not be suitable for children or other specific groups, limiting their applicability and effectiveness in these populations. Furthermore, cases involving multiple coagulopathies or patients who have received various treatments (e.g., anticoagulants, fibrinolytics) can complicate the assessment of the patient's actual coagulation status, potentially resulting in mismanagement of the condition [24].

Emerging trends and future directions

Emerging trends and future directions in the scoring and diagnosis of DIC focus on enhancing diagnostic accuracy and clinical utility through refinements to existing scoring systems and integrating novel biomarkers and imaging techniques [25]. Recent modifications to DIC scoring systems aim to improve overt and non-overt DIC identification. The JSTH has introduced a diagnostic algorithm that considers the underlying pathology, whether primary, hematopoietic, or infectious, allowing for a more tailored approach to scoring. This updated system incorporates additional parameters such as antithrombin levels and specific fibrin degradation products, offering a more comprehensive assessment of coagulation status [26]. Including hemostatic markers, such as protein C activity and plasminogen activator inhibitor 1, into scoring systems has shown promise in enhancing prognostic value. Studies suggest that these markers can significantly improve mortality predictions in patients with severe sepsis and DIC, with sensitivity rates reaching up to 84.6%. This trend toward integrating biochemical markers into scoring reflects a broader shift toward precision medicine in managing DIC [27].

Emerging research is identifying potential biomarkers that could further refine DIC diagnosis. For example, thrombin-antithrombin complexes (TAT) and soluble fibrin are being investigated for their ability to provide additional insights into the coagulation process and DIC severity. Incorporating these biomarkers into scoring systems could enhance diagnostic accuracy and risk stratification [28]. While traditional laboratory tests remain fundamental for DIC diagnosis, advanced imaging techniques are being explored for their potential utility. Ultrasound and magnetic resonance imaging (MRI) could assess microvascular thrombosis and bleeding complications associated with DIC. These techniques may offer real-time insights into patients' hemodynamic status and guide therapeutic interventions more effectively [29]. The future of DIC diagnosis is set to advance significantly through the refinement of scoring systems and the integration of novel biomarkers and imaging techniques. These developments aim to enhance diagnostic accuracy and patient stratification, ultimately optimizing clinical outcomes and tailoring treatment strategies. Ongoing research and validation of these approaches will be crucial in shaping the future landscape of DIC management [30].

## Conclusions

In conclusion, the accurate diagnosis and effective management of DIC hinge significantly on using reliable scoring systems. This review has highlighted the strengths and limitations of various scoring systems, including the ISTH, JAAM, and other regional criteria. While each system offers valuable insights into the severity and progression of DIC, their effectiveness can vary based on clinical context and patient population. The timely application of these scoring tools can substantially impact patient outcomes by guiding appropriate interventions and treatment strategies. However, challenges remain in standardizing these systems and addressing their limitations. Future research should focus on refining these scoring methods, exploring novel biomarkers, and integrating advanced diagnostic technologies to enhance DIC assessment's accuracy and clinical utility. By advancing our understanding and application of DIC scoring systems, we can improve diagnostic precision and patient management, ultimately leading to better clinical outcomes.
